# Discovery of a New Neisseria gonorrhoeae Type IV Pilus Assembly Factor, TfpC

**DOI:** 10.1128/mBio.02528-20

**Published:** 2020-10-27

**Authors:** Linda I. Hu, Shaohui Yin, Egon A. Ozer, Lee Sewell, Saima Rehman, James A. Garnett, H Steven Seifert

**Affiliations:** a Department of Microbiology-Immunology, Northwestern University Feinberg School of Medicine, Chicago, Illinois, USA; b Division of Infectious Diseases, Northwestern University Feinberg School of Medicine, Chicago, Illinois, USA; c Centre for Host-Microbiome Interactions, Dental Institute, King’s College London, London, United Kingdom; The Ohio State University School of Medicine

**Keywords:** *Neisseria gonorrhoeae*, pili, pilus assembly, type IV pili

## Abstract

Neisseria gonorrhoeae relies on type IV pili (T4p) to promote colonization of their human host and to cause the sexually transmitted infection gonorrhea. This organelle cycles through a process of extension and retraction back into the bacterial cell. Through a genetic screen, we identified the NGO0783 locus of N. gonorrhoeae strain FA1090 as containing a gene encoding a protein required to stabilize the type IV pilus in its extended, nonretracted conformation. We have named the gene *tfpC* and the protein TfpC. Deletion of *tfpC* produces a nonpiliated colony morphology, and immuno-transmission electron microscopy confirms that the pili are lost in the Δ*tfpC* mutant, although there is some pilin detected near the bacterial cell surface. A copy of the *tfpC* gene expressed from a *lac* promoter restores pilus expression and related phenotypes. A Δ*tfpC* mutant shows reduced levels of pilin protein, but complementation with a *tfpC* gene restored pilin to normal levels. Bioinformatic searches show that there are orthologues in numerous bacterial species, but not all type IV pilin-expressing bacteria contain orthologous genes. Coevolution and nuclear magnetic resonance (NMR) analysis indicates that TfpC contains an N-terminal transmembrane helix, a substantial extended/unstructured region, and a highly charged C-terminal coiled-coil domain.

## Abstract (precis)

Most bacterial species express one or more extracellular organelles called pili/fimbriae that are required for many properties of each bacterial cell. The Neisseria gonorrhoeae type IV pilus is a major virulence and colonization factor for the sexually transmitted infection gonorrhea. We have discovered a new protein of Neisseria gonorrhoeae called TfpC that is required to maintain type IV pili on the bacterial cell surface. There are similar proteins found in other members of the *Neisseria* genus and many other bacterial species important for human health.

## INTRODUCTION

Neisseria gonorrhoeae is the main causative agent of the sexually transmitted infection gonorrhea. There were 555,608 reported cases of gonorrhea reported in the United States in 2017 and an estimated 86.9 million worldwide, as well as an alarming rise in antibiotic resistance (1, 2). There are three major problems that complicate the treatment of gonorrhea. First, the rapid rise of antibiotic resistance has resulted in strains that are refractory to conventional treatments (3). Second, many patients are asymptomatic, remain untreated, and contribute to the spread of the disease. Third, infection does not result in long-term immunity to reinfection. These attributes have made a vaccine or novel antimicrobials desirable, but to date there are no viable novel treatments. The uncertainty for future treatment options emphasizes the need for new knowledge about N. gonorrhoeae colonization and pathogenesis and innovative modes of treatment.

Almost all Gram-negative bacteria and a subset of Gram-positive bacteria express type IV pili (T4p) (4). There are three major subsets of T4p, and there is a clear evolutionarily relationship with type II secretion systems (T2S) and archaeal flagella (5). T4p provide a wide range of phenotypes to the organisms that express them and are important organelles that promote bacterial colonization and pathogenesis. N. gonorrhoeae T4p are the only known virulence factor absolutely required for colonization (6–8). The type I T4p of Neisseria meningitidis are closely related to the N. gonorrhoeae T4p and are also necessary for colonization and disease (9).

T4p have multiple functions that are critical for N. gonorrhoeae pathogenesis. The pilus is an essential factor for colonization, enhancing the ability of the bacterium to adhere to and interact with host cells and tissues at infection sites (10). The pilus is also required to promote bacterium-bacterium interactions and the formation and dissolution of microcolonies and biofilms (11). T4p are required for twitching motility, a specialized form of locomotion that requires T4p retraction (9), to enhance bacterial interactions with the epithelium (12). The T4p apparatus is a bidirectional secretion apparatus that engages in pilus secretion, importing DNA for genetic transformation and the spread of antibiotic resistance, importing the pilus for twitching motility, and importing other molecules like antibiotics (13). T4p expression greatly increases gonococcal (GC) resistance to the oxidative and nonoxidative killing mechanisms of polymorphonuclear leukocytes (PMNs) (14). While we have a grasp of many molecular mechanisms underlying *Neisseria* T4p assembly and function, many questions remain about how this dynamic fiber functions in pathogenesis.

Several proteins are involved in the assembly and function of T4p. The main pilin subunit, PilE, starts as a prepilin with a 7-amino-acid leader sequence. After secretion through the inner membrane (IM), the leader sequence is cleaved by the PilD signal peptidase to produce the mature protein (15). PilD is also required to process the minor pilins that share N-terminal amino acid sequence similarity with pilin (16, 17). The PilQ protein is of the secretin class and forms a pore through the outer membrane (18, 19). The PilC protein (20) is localized to the outer membrane and has been implicated in contributing to adherence and modulating pilus retraction (21, 22). PilC is also reported as being localized to the pilus tip (23). PilP and PilW have been shown to interact with PilQ (24). The minor pilin proteins PilH to PilL are proposed to prime pilus assembly within the periplasm (25). The minor pilins PilV and ComP are dispensable for pilus assembly but have specific roles in adherence and transformation (16, 17). The PilF (also known as PilB), PilT, and PilU proteins are cytoplasmic NTPases involved in modulating pilus extension and retraction (15, 26, 27).

We previously demonstrated that the activity of the Mpg zinc metalloprotease is required to maintain T4p exposed on the bacterial cell surface (14). We also showed that Mpg activity on T4p mediates protection from both oxidative and nonoxidative killing mechanisms of PMNs (14, 28). The increased sensitivity of nonpiliated cells to oxidative and nonoxidative killing is phenocopied by nonpiliated N. gonorrhoeae sensitivity to the iron-dependent antimicrobial compound streptonigrin (SNG). A transposon insertion sequencing (InSeq) screen for mutants that alter SNG sensitivity revealed a new T4p assembly factor that we named TfpC.

## RESULTS

We conducted a saturating InSeq screen of GC strain FA1090 to identify genes that when inactivated provided decreased or increased survival to SNG lethality. We will report the rationale and full results of the InSeq SNG screen elsewhere. From this screen, we identified the NGO0783 locus of N. gonorrhoeae strain FA1090 as providing an average 38.7-fold decrease in representation under 0.4 μM SNG selection when interrupted with any of nine distinct transposon insertion sites within the open reading frame (ORF).

**FIG 1:**
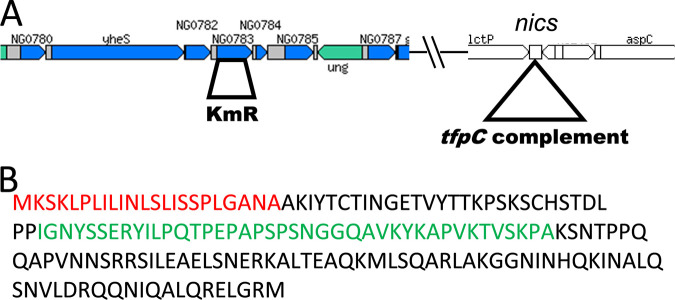
Cartoon of the NGO0783 locus, mutants, and complements. (A) Cartoon of the chromosomal regions of strain FA1090 with the NGO0783 locus and the surrounding loci and the location of the *Neisseria* chromosomal complementation (*nics*) site (58) expressing the *tfpC*::*flag* complement. (Reproduced from http://stdgen.northwestern.edu with permission). (B) Predicted amino acid sequence of the N. gonorrhoeae TfpC protein. The cleavable signal sequence is shown in red. The disordered hydrophobic region is shown in green.

**FIG 2:**
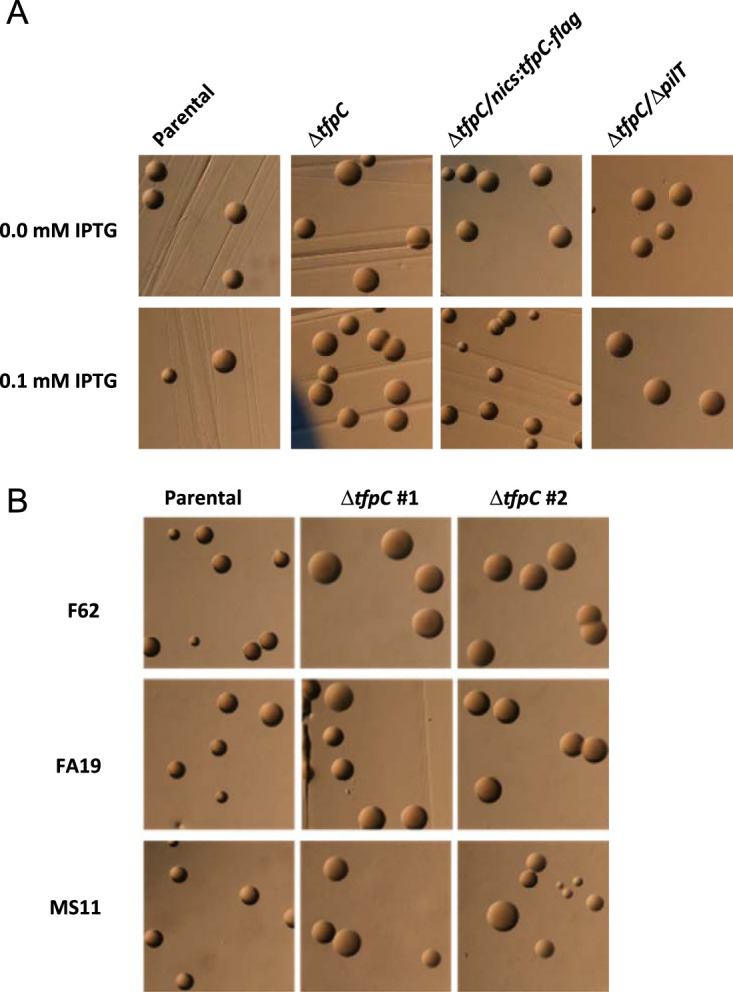
Analysis of pilus-dependent colony morphology. (A) Stereo-micrograph pictures of 22-h N. gonorrhoeae colonies grown with or without 0.1 mM IPTG in the medium. The parental strain is FA1090 1-81-S2 *recA6*. (B) Pilus-dependent colony morphology changes in two different Δ*tfpC* transformants of N. gonorrhoeae strains F62, FA19, and MS11 grown for 22 h. Piliated colonies (e.g., the parental strain) are small, have a dark ring at the edge of the colony, and are domed. Nonpiliated colonies (e.g., the Δ*tfpC* strain) are larger, have no dark ring or a less pronounced ring, and are flatter.

We constructed a loss-of-function mutation of the NGO0783 locus (Fig. 1A) in strain FA1090 by deleting most of the open reading frame and inserting a nonpolar (Table 1) kanamycin (Kan) resistance (Km^r^) gene. The Δ*tfpC* mutant showed a characteristic P-colony morphology, with flatter colonies with more spreading that results in larger-diameter colonies than in the *P*^+^ parent without the *P*^+^ dark edge (Fig. 2A) (6). These changes in colony morphology are consistent with a reduction in pilus expression (29) or an effect on pilus bundling (30). We introduced the Δ*tfpC* mutation into three other N. gonorrhoeae isolates, and in each strain, this mutation resulted in P-colony morphology (Fig. 2B). The mutant also showed reduced transformation competence (5.6 × 10^−5^ transformants/CFU versus 1.3 × 10^−3^ transformants/CFU for the piliated parent), consistent with its nonpiliated colony morphology, but the NGO0783 mutant is more competent for DNA transformation than a Δ*pilE* mutant that does not transform under these conditions (<8 × 10^−7^ transformants/CFU). This phenotype is similar to those of other N. gonorrhoeae mutants that can still assemble pili but cannot maintain them in an extended conformation (14, 25, 31). However, unlike with those mutants, introducing a *pilT* loss-of-function mutation to the NGO0783 mutant did not restore the parental *P*^+^ colony morphology (Fig. 2A).

**TABLE 1:** Effect of the Km^r^ gene insertion into the Δ*tfpC* strain (in the NGO0783 locus) on surrounding genes in the operon as determined by quantitative RT-PCR

Locus	Fold change
NGO_0779	1.1
NGO_0780	2.4
NGO_0781	2
NGO_0782	2.7
NGO_0784	2.2
NGO_0785	2.4

We introduced a series of isopropyl-d-1-thiogalactopyranoside (IPTG)-regulated complementation constructs at an ectopic locus in the FA1090 chromosome to express native TfpC, as well as an epitope-tagged (Flag-tagged) version (Fig. 1A). The IPTG-regulated *tfpC-flag* complement construct restored a piliated colony morphology (Fig. 2A) and SNG resistance (not shown). We used the Flag-tagged complement for all further analysis. Based on these preliminary results, we predict that this gene is involved in T4p elaboration and therefore named the gene within the NGO0783 locus *tfpC* for type four pilus assembly protein C, and we named the protein TfpC. We cannot rule out other roles for the TfpC protein in cellular processes distinct from piliation but did not observe any obvious cellular phenotypes that suggest an alternative function.

**FIG 3:**
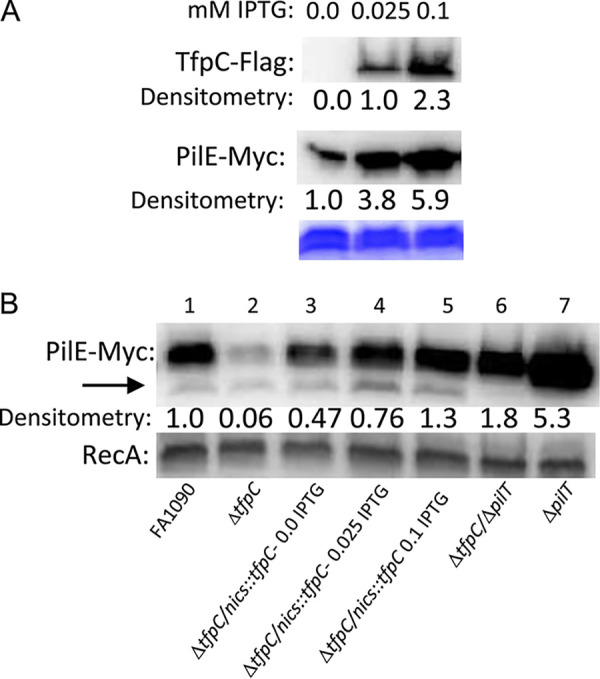
Western blot analysis of total TfpC and pilin expression. (A) Strain FA1090 1-81-S2 *pilE-myc* Δ*tfpC/nics*::*tfpC-flag* was grown with different levels of the IPTG probe, and whole-cell lysates were probed with anti-FLAG monoclonal antibody (MAb) or the anti-Myc MAb. The section of the Coomassie blue-stained gel shows equal loadings of the proteins in the replicate gels. Estimates of relative protein amounts as determined by densitometry are shown below each blot. A representative Western blot of three independent repeats is shown. (B) The Δ*tfpC*, Δ*tfpC/nics*::*tfpC-flag*, Δ*tfpC*/Δ*pilT*, and Δ*pilT* strains in the FA1090 1-81-S2 myc-tagged *pilE* background (Q155) were grown with different levels of IPTG, and whole-cell lysates were probed with anti-Myc MAb. After development, the blot was washed and reprobed with anti-RecA antisera (E. coli). A Western blot of two independent repeats is shown. We presume that the smaller band indicated by the arrow is the truncated pilin form S-pilin (59).

Wild-type levels of *tfpC* mRNA were produced from the complemented strain when 0.025 mM IPTG was added to the growth medium (Table 2). Western blot analysis of TfpC protein levels confirmed the quantitative real-time PCR (Q-RT-RCR) results (Fig. 3A). Analysis of total pilin levels with pilin tagged with a c-Myc epitope tag (32) showed that the Δ*tfpC* mutant had lower levels of pilin protein than the parental FA1090 strain, which were restored when the complemented strain was grown with 0.025 mM IPTG in the growth medium (Fig. 3B). Moreover, 0.1 mM IPTG produced 3.7-fold-higher levels of *tfpC* mRNA (Table 1). Even with overexpression of *tfpC*, there was no noticeable growth (data not shown) or colony morphology phenotype (Fig. 2A) compared to those of the parental strain. When we grew the complemented strain with 0.1 mM IPTG in the medium, there was a small increase in the pilin protein band relative to that in the parental strain. Surprisingly, a Δ*tfpC* Δ*pilT* double mutant had more total pilin protein by Western blotting than the Δ*tfpC* mutant alone. These results show that TfpC acts to stabilize pilin, the loss of the pilus in the Δ*tfpC* mutant is dependent on PilT, and the absence of pilus retraction stabilizes pilin in both wild-type TfpC-expressing strains and the Δ*tfpC* mutant background.

**TABLE 2:** Fold change in *tfpC* expression in response to IPTG in the growth medium

IPTG concn (mM)	Fold change in *tfpC* expression in the Δ*tfpC*/*nics*::*tfpC* mutant
0	0.15
0.01	0.32
0.02	0.65
0.025	1.24
0.03	1.75
0.05	3.1
0.1	3.7
0.5	7.3
1	7.1

**FIG 4:**
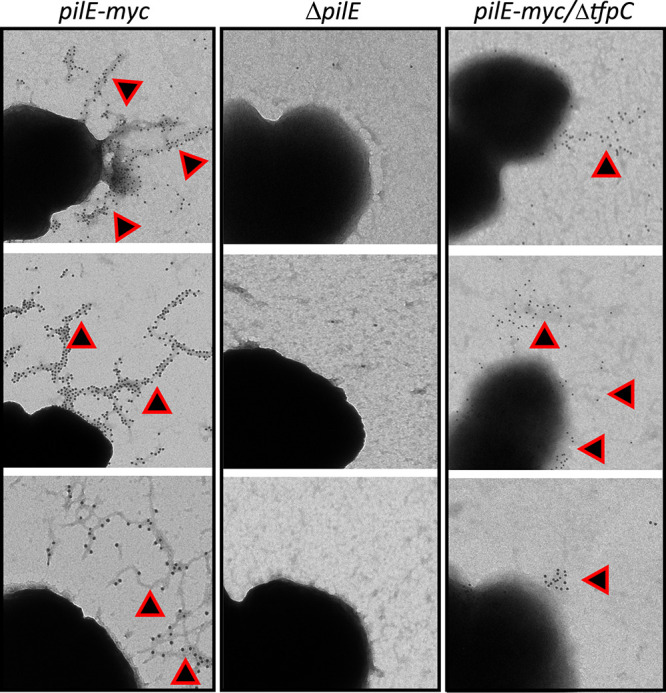
Immuno-TEMs of pilus expression on the Δ*tfpC* mutant. Micrographs of cells lifted onto grids from 22-h colonies of the FA1090 *pilE-myc* strain, the FA1090 Δ*pilE* nonpiliated mutant, and the FA1090 *pilE-myc* Δ*tfpC* mutant, which were reacted with anti-Mac MAb and then secondary gold-labeled with anti-mouse IgG. The small round gold particles show where immune-reactive pilin is localized and are indicated with triangles. These are representative images from two independent experiments.

**FIG 5:**
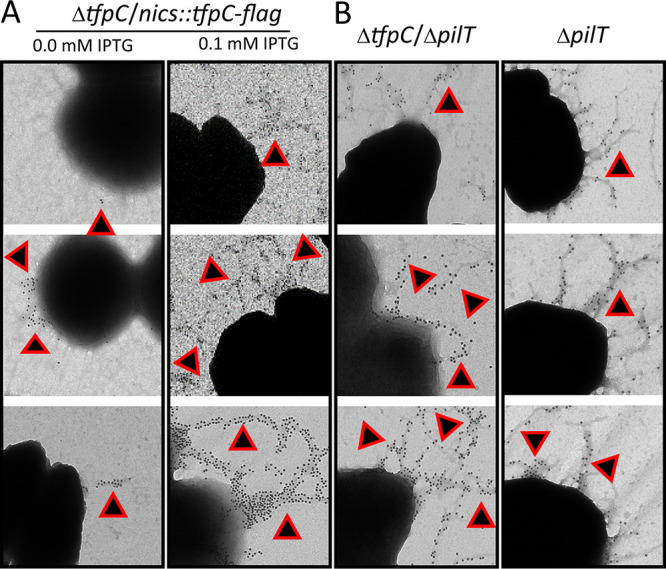
Immuno-TEMs of pilus expression. (A and B) Representative micrographs of cells lifted onto grids from 22-h colonies of the FA1090 *pilE-myc* strain and the Δ*tfpC*/*nics*::*tfpC-flag* strain grown with or without IPTG to induce TfpC expression (A) and the FA1090 *pilE-myc* Δ*tfpC* Δ*pilT* and FA1090 *pilE-myc* Δ*pilT* strains, which were reacted with anti-Mac MAb and then secondarily gold labeled with anti-mouse IgG (B). The small round gold particles show where immune-reactive pilin is localized and are highlighted with triangles. These are representative images from two independent experiments.

We determined the effect of the Δ*tfpC* mutation on piliation in FA1090 using the c-Myc epitope-tagged *pilE* (32) to allow visualization of the pilus using gold-labeled secondary antibody in immuno-transmission electron micrographs (IM-TEMs) (Fig. 4 and 5). These types of electron micrographs have limitations since they cannot quantitate pili because the bacterial cells are absorbed from a bacterial colony onto the grid. However, the IM-TEMs showed that the Δ*tfpC* mutant lost piliation (Fig. 4) and that Flag-tagged *tfpC* restored pili in the complemented strain (Fig. 5). These results were consistent with the colony morphology phenotypes. Interestingly, many of the Δ*tfpC* mutant cells still had antibody binding near the bacterial cell surface (Fig. 4), which was not observed with a nonpiliated *pilE* mutant strain. This result suggested that there were short pili on the cell surface or another form of pilin that reacts with the antibody near the cell surface. The IM-TEM analysis of the Δ*tfpC* Δ*pilT* double mutant showed that loss of PilT restored pilus expression to the Δ*tfpC* mutant (Fig. 5), a result consistent with the Western blot analyses (Fig. 3). However, the pili in the Δ*tfpC* Δ*pilT* double mutant did not show any essential differences from those expressed on the parental strain.

**FIG 6:**
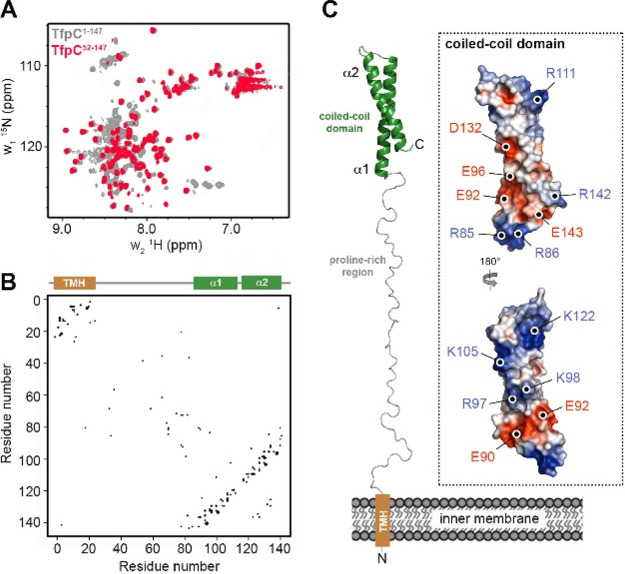
Structural model of TfpC. (A) Overlay of ^1^H-^15^N HSQC NMR spectra for mature TfpC (residues 1 to 147, gray) and N-terminally truncated TfpC (residues 52 to 147, red). Proton resonances observed between ∼8.0 and 8.5 ppm indicate the presence of characteristic clusters of unstructured backbone amides. The peaks resonating at high chemical shifts (>8.5 ppm) correspond to highly ordered backbone amides present in secondary-structure elements. However, a lack of dispersion (no peaks were >9.0 ppm) suggests the presence of an extended helix or coiled-coil structure. Removal of N-terminal residues from TfpC results in a reduction of disordered resonances. (B) Coevolution contact map for TfpC with secondary-structure features highlighted. The transmembrane helix (TMH) is brown, and helices are green. (C) Coevolution coupling-restrained model of TfpC. The inset shows the C-terminal coiled-coil domain as the electrostatic surface potential, with charged surface residues highlighted. The surface of the C-terminal domain is composed of both large positive and negative patches, which may mediate recognition of a partner protein(s).

Bioinformatic analysis indicated that TfpC has a cleavable periplasmic localization signal at its N terminus, followed by a short transmembrane helix, an extended proline-rich region, and a helical domain at the C terminus (Fig. 1B). This predicted structure was supported by nuclear magnetic resonance (NMR) experiments where we compared ^1^H-^15^N heteronuclear single quantum coherence (HSQC) spectra for mature recombinant TfpC (residues 1 to 147, minus the signal sequence) and an N-terminally truncated TfpC (residues 52 to 147) (Fig. 6A). Proton resonances for the N-terminal region of TfpC were observed between ∼8.0 and 8.5 ppm, indicative of unstructured peptide, while highly ordered backbone amide peaks (>8.5 ppm) did not extend above 9.0 ppm, which suggested the presence of an extended helix or coiled-coil structure at the C terminus.

Analysis of the coevolution between different amino acid sites within a protein sequence can provide strong evidence for interresidue interactions, such as those found in protein subdomains (33). We therefore performed coevolution analysis on the mature TfpC sequence using the EVcouplings Python framework (34). We identified 1,065 similar sequences and used them in the alignment stage, which provided an excellent alignment solution with a ratio of effective sequences to protein length of 5.15 (Fig. 6B). There were 77 strong evolutionary couplings identified, which generally clustered between residues located either in the transmembrane helix region or the C-terminal helical domain, but very few couplings were observed in the extended central region. We then used these couplings as distance restraints to generate a model of TfpC. The model suggests that the N terminus of TfpC may insert into the bacterial inner membrane, while a C-terminal coiled-coil domain is projected into the periplasm via an extended proline-rich region (Fig. 6C). The C-terminal domain contains a high proportion of charged residues, and the surface of the model is composed of both large positive and negative patches. This indicates that this region may be involved in the recognition of a partner protein(s), presumably in the periplasm, and that electrostatic interactions drive important interactions with the pilus machinery (Fig. 6C).

## DISCUSSION

Almost every Gram-negative bacterial species expresses at least one Tfp protein, as do many Gram-positive organisms. The apparatus that allows the expression and function of T4p spans the bacterial envelope and is evolutionarily related to the T2S apparatus in many bacterial species and to archaeal flagella. The assembly and function of these organelles have been studied intensively in these disparate types of prokaryotes. Since the InSeq transposon screen was to identify gene products involved in resistance or sensitivity to streptonigrin, we were intrigued when we found that the NGO0783 locus contained a gene product important for piliation.

Bioinformatic analysis of the open reading frame in the NGO0783 locus provided several predictions about the protein structure and function. The predicted protein has a predicted molecular weight of 18.455 kDa and a basic pI of 10.8. The TfpC protein has a standard, Sec-dependent, cleavable signal sequence (probability = 0.99 by SignalP 5.0), and the mature protein has a hydrophobic N terminus with many proline residues (Fig. 1B). The best-fit structural prediction model from Phyre2 is an HR1 repeat protein with regions connected by a central hinge (Fig. 5). There is enrichment of the TfpC protein in cell envelopes and membrane vesicles when the MlaA phospholipid removal protein is inactivated (35), showing that TfpC is localized to the bacterial envelope. The TfpC protein sequence is 99 to 100% conserved in all sequenced N. gonorrhoeae isolates, suggesting that it is not surface exposed. Based on these analyses, we predict that this ORF localizes to the bacterial periplasm.

BLASTP revealed that the ORF has a DUF4124/pfam13511 domain of unknown function and is the only member of the cl16293 superfamily of proteins. There are orthologues of TfpC present in genomic sequences of Neisseria meningitidis, Neisseria lactamica, Neisseria polysaccharea, and Neisseria cinerea with 100% amino acid identity. There were also other *Neisseria* sp. orthologues with lower but significant similarity, including several with an N-terminal extension and an additional middle domain not found in in the N. gonorrhoeae orthologue. A search of the Pfam database shows 948 bacterial species in many genera with proteins with the DF13511 (DUF4124) domain; however, how many of these proteins are true orthologues and involved in T4p or T2S is not known from this type of analysis. In our searches, we found the Dsui_1049 locus of the bacterium Dechlorosoma suillum PS (an environmental Gram-negative strain also called *Azospira* [36; https://lpsn.dsmz.de/species/azospira-oryzae]) that shows a Waterman_Eggert score of 169 and an E value of <5.6e–10 with TfpC. Many of the genes in *D. suillum* PS that show a fitness correlation with a Dsui 1049 mutant are T4p-associated genes, supporting a broad role for TfpC orthologues in piliation (Fitness Browser, http://fit.genomics.lbl.gov). Interestingly, there are other well-studied T4p-expressing species with no close orthologues, such as Pseudomonas aeruginosa and Vibrio cholerae. These species do have DUF4124 domain proteins, but there is too limited sequence similarity to assign these as orthologues. It will be interesting to determine why only some species that express T4p have a TfpC orthologue and whether the more distant orthologues are all involved in T4p expression or may alternatively be involved in T2S or other related processes.

Introducing a Δ*pilT* loss-of-function mutation into the Δ*tpfC* strain produced two contrasting phenotypes. The inactivation of pilus retraction through loss of PilT did not restore the piliated colony morphology (Fig. 2A), but the TEMs clearly showed that pili where restored when PilT was inactivated and there was no observable difference between the parental pili and the pili observed with the Δ*tpfC* Δ*pilT* mutant (Fig. 5B). We assume that the pili expressed on the Δ*tpfC* Δ*pilT* mutant are different in a way that alters the colony morphology that is not reflected in the TEMs.

One of the more interesting phenotypes of the Δ*tfpC* mutant is the loss of the pilin protein in the mutant and the stabilization of pilin when we overexpressed TfpC with 0.1 mM IPTG (Fig. 3). The observation that loss of pilus retraction in the Δ*tfpC* Δ*pilT* double mutant also stabilizes pilin suggests that the role of TfpC in stabilizing pilin occurs after pilus retraction and not during pilus extension or within the extended fiber. However, the fact that a Δ*pilT* mutant strain with wild-type *tfpC* also shows a stronger pilin band suggests that PilT-dependent pilin degradation occurs all the time. This observation of a retraction-dependent destabilization of pilin has been previously reported for strain MS11 (37). We propose that pilin that is within the assembled pilus fiber is protected from proteolysis but that, upon retraction, pilin becomes exposed to periplasmic proteases. In the future, determining whether proteolysis occurs during the process of retraction or after pilin returns to the cytoplasmic membrane will provide important insight into T4p dynamics.

Based on the phenotypes of the Δ*tfpC* mutant and Δ*tfpC* Δ*pilT* double mutant, we propose that the TfpC protein is not necessary for T4p expression but rather is necessary to maintain the T4p in an extended state until retraction occurs. We speculate that the surface-associated pilin detected in the Δ*tfpC* mutant (Fig. 4) may be pili caught in the process of retraction. This same PilT-dependent loss of pilus expression occurs when several other pilus-associated proteins are inactivated, and we have proposed that there might be a peptidoglycan-linked antiretraction complex that mediates this phenotype since mutants lacking several peptidoglycan-modifying enzymes (Mpg and DacB/C) also show a PilT-dependent modulation of pilus expression (14, 38). There are other mechanisms that may account for this phenotype, such as a role of TfpC and other proteins in modulating PilT activity, acting through the inner membrane complex. Determination of the precise subcellular localization of TfpC and its interaction partners will be required in future work to devise the mechanisms of TfpC in modulating pilus dynamics.

## MATERIALS AND METHODS

### Strains and growth.

The studies performed here used mainly N. gonorrhoeae strain FA1090 PilE variant 1-81-S2 (39) and its isogenic derivatives. Strains MS11, F62, and FA19 were also tested (Table 3). The sequence of *pilE* was confirmed to be 1-81-S2 using PCR and sequencing with primers pilRBS and SP3A (Table 4). N. gonorrhoeae was grown in GC medium base (Difco) plus Kellogg supplements I and II [22.2 mM glucose, 0.68 glutamine, 0.45 mM cocarboxylase, 1.23 mM Fe(NO3)3] (GCB) at 37°C in 5% CO_2_. Antibiotics and their concentrations used for selection in GCB were kanamycin (Kan) at 50 μg/ml and erythromycin (Erm) at 2 μg/ml. E. coli strains One Shot TOP10 Electrocomp E. coli (Invitrogen), DH5α, and BL21(DE3) (New England Biolabs), used to propagate plasmids or protein, were grown in solid Luria-Bertani (LB) medium containing 15 g/liter agar or liquid medium at 37°C. The antibiotic and its concentrations used in LB medium were ampicillin (Amp) at 100 μg/ml. Oligonucleotides used are listed in Table 5.

**TABLE 3:** N. gonorrhoeae strains and plasmids^*a*^

Strain or plasmid	Description	Reference/source
Strains		
N-1-1	FA1090 1-81-S2 PilE variant	39
N-1-14	FA1090 1-81-S2 PilE variant, *recA6* Tet^r^	32
N-1-60	FA1090 multisite G4 mutant 1-81-S2 *pilE* variant, *pilC1*_PL_	This study
N-1-69	An unmarked Δ*pilE* mutant (deletion from the 6th amino acid to the stop codon in *pilE* from Alison Criss) in N-1-60	This study
N-3-3	Δ*tfpC*::*kan* in N-1-60 Kan^r^	This study
K-16-47	FA1090 1-81-S2 *recA6 pilE-myc* Tet^r^ Cam^r^	This study
Q155	FA1090 1-81-S2 *pilE-myc* Cam^r^	This study
Q115	Δ*tfpC*::*kan* in K-16-47, Kan^r^ Tet^r^ Cam^r^	This study
Q165	Δ*tfpC*::*kan* in Q155, Kan^r^ Cam^r^	This study
J-11-20	FA1090 Δ*pilT*::*ermC* Erm^r^	52
MS11	Piliated strain	Lab stock
F62	Piliated strain	Lab stock
FA19	Piliated strain	Lab stock

Plasmids		
pTwist-Δ*tfpC*::*kan*	pTwistAmpMC plasmid carrying a synthetic Δ*tfpC*::*kan* construct, Amp^r^ Kan^r^	This study
pGCC4	IPTG-inducible *Neisseria* chromosomal complementation (*nics*) vector, Erm^r^ Kan^r^	47
pGCC2	*nics* vector, Erm^r^	49
K-20-71	c-Myc-tagged *pilE* (*pilE-myc*), Cam^r^	32

a Tet^r^, tetracycline resistance; Cam^r^, chloramphenicol resistance; Amp^r^, ampicillin resistance.

**TABLE 4:** Synthetic genes

Gene	Description	Sequence (5′ to 3′)^*a*^
*tpfC*::PacI-His-NotI	Used to generate the Δ*tfpC*::*kan* strain	TCGTGTGCCGATGCTGATTTACCTAAAATCAGCAGCCATCAGGGAGGCGGATACCGCCTGAAAATTAAAAAACTTAGTCAAGAAGCCAAAATACACACAGGAAACAAAAAGAAAAACAAAAAACATGCCGGGAAAAAGAACAGACAGGCTGCCAAAGCCCCGAAGGAAAATCAAAAATAAACAACCGAAAAGAAAAGCCCATAAAACGCCAAGAAAACCTTACAAAAAATCCTCAAAAAATCAAATTATTATCCGAATATCAAACACATTATGAAATCAAAACTCCCCTTAATCCTAATCTTAATTAA**G***TACCCATACGATGTTCCAGATTACGCT*GCGGCCGC**G**GCACTGCAAAGAGAATTGGGACGTATGTAAGGCCGTGTTTTCAAATCGACCGTTCCAAGGATTTGACAGAAGAAATGATGAAAAAGCAGGAGAATTTTTGGGATAAGTTGGGCGATTTACTGTTTGCGCCCGTTGATATAATGTTTTGGATTAAAAAAGTATGGGCGGCATATCCTGTGTGTCGGCTGCCTGTAATCGTATTGAAGGTCAACGTATTCCCCAATACCGGCTACACGTACAACTGTTTCCGATAGTTCGCATAATGTATATTATGTTAAATTTATAATGGATTGAATAGAT
*tfpC*	Full-length TfpC	CCATGGTCCATCACCATCATCATCATGTTGACGATGATGATAAAATGGCCAAGATTTATACCTGCACCATTAACGGTGAAACCGTGTATACCACCAAACCGAGCAAAAGCTGTCATAGCACCGATCTGCCTCCGATTGGTAATTATAGCAGCGAACGTTATATTCTGCCGCAGACACCGGAACCGGCACCGAGTCCGAGCAATGGTGGTCAGGCAGTTAAATACAAAGCACCGGTTAAAACCGTTAGCAAACCTGCAAAAAGCAATACCCCTCCGCAGCAGGCACCGGTGAATAATAGCCGTCGTAGCATTCTGGAAGCAGAACTGAGCAATGAACGTAAAGCACTGACCGAAGCACAGAAAATGCTGAGCCAGGCACGTCTGGCAAAAGGTGGTAACATTAATCATCAGAAAATTAACGCCCTGCAGAGCAATGTTCTGGATCGTCAGCAGAATATTCAGGCACTGCAGCGTGAACTGGGTCGTATGTAACTCGAG
Multisite G4	Carries multiple mutations in the G4 sequence	atgccgtctgaaTGAACCAACTGCCACCTAAGGCAAATTAGGCCTTAAATTTCAAATAAATCAAACGGTAAGTGATTTTCCACGGCCGCCCGGATCAACCCGGGCGGCTTGTCTTTTAAGGGTTTGCAAGGCGGGCGGGGTCGTCCGTTCCGAAGCCATCCTTTTGGCCGAAGGTCAAAAATCAGCCGTTACCGGGTATTGCCCGAATCACGGCATATGGCCGGAAAACTTCGTCATTCCCGCGAAAGCGGGAATCTAGGTCTGTCGGCACGGAAACTTATCGGGTAAAAAGGTTTCTCCGGTCCTGAGTCCTGGATTCCCACTTTCGTGGGAATGACGGGATTTAATGATGCCGCCGGCAACGAAAAAATCGAAACCAAGCACCTGCCGTCAACCTGCCGCGACGCTTCATCTGCCGGTTGCATAGAAACACCACGCGCCGATTTCAAATGCTTTCCAAGAAAACGGAGCTTTTTAAAAAATAAAAAATTCCCCACCACACCCCCACTATTCTAACGCGTAAATTCAAAAATCTCAAATTCCGACCCAATCAACACACCCGATACCCCATGCCAATAAAAAAGTAACGAAAATCGGCACTAAAACTGACAATTTTCGACACTGCCGCCCCCTACTTCCGCAAACCACACCCACCTAAAAGAAAATACAAAATAAAAACAATTATATAGAGATAAACGCATAAAATTTCACCTCAAAACATAAAATCGGCACGAATCTTGCTTTATAATACGCAgTTGTCGCAACAAAAAACCGATGGTTAAATACATTGCATGATGCCGATGGCGTAAGC
*pilC1*_PL_	Carries *pilC1*_PL_ allele	atgccgtctgaaCAAACGGTTGCGGATTGCCAAAAACCGCTGTACCATGGATAAGCGCGCAAGGAGAATGATGCGGCAACCCTATACATTGCACCCCGTCAGAGGGGCGCGTTACCTTTGCGAACATCCCCCTTTGGCAGCCGGGCGAAGGGGGCTTTGCAACCGGCAATCCGGCGGGCGCGGGATCGGGCGGTTTGCCGAATCCCGCCGTTTGCGGCGCGCCTGCCGCCGACGGTATCCCGCGAAGCAAGATTTAAGGGATAAAATATGTTCCAACACGCAGGGCGGCACATAAGGCGCCGCCCTGATTCGGAAGGGCTTGCACCCCTCCCGGACAAAGCCTGATCCTGCCGTCCCGAAGGACGGATGTCCGAGCGGCGGGGTTTCAACCGAAAAGGAAATACGATGAATAAAACTTTAAAAAGGCGGGTTTTCCGCCATACCGCGCTTTATGCCGCCATCTTGATGTTTTCCCATACCGGCGGAGGTGGCGGGGCCCAGGCGCAAACGCAAACCCGTAAATACGCTATTATCATGGACGAACGAAATCAGCCGGAGGTAAAGTGGGAGGGTTCATATTCAACCTTAAGGGAAAAAGACGGGGAACGCAAATTTATCTATACGAACCAGAGAAACAAGTTGAACCAACAAAACAATTTTATCTCATTCGACAATACCGATACCCTTGTTTCCCGACAAAGCGGTACTGCCGTTTTTGGCACAGCCACCTACCTGCCGCCCTACGGCAAGGTTTCCGGCTTTGATACCGCCGAGCTGAACAAGCGCGGCAATGCCGTCAATTGGATTCATACCACCCGGGCCGGGCTGGCAGGCTACGTCTACACCGGCGTCATATGCAGAGACACAGGGCAATGCCCCCAACTTGTCTATAAAACCCGATTTTCCTTCGACAACACCGGTTTGGCAAAAAATACGGGCAGGCTGGATAGGCACACAGA

a Restriction sites are underlined, the HA tag is italicized, an additional nucleotide to keep the reading frame is in bold, a mutated G4 or *pilC1*_PL_ sequence is shaded, and the additional DNA uptake sequence is lowercase.

**TABLE 5:** Oligonucleotides

Name	Sequence (5′ to 3′)	Reference/source
PacI_nptII181_F	ACTGTTAATTAAATGGCGATAGCTAGACTGGG	This study
DUS12_nptIIR	ATGCCGTCTGAAATTTCGAACCCCAGAGTCC	This study
NotI_DUS12	ACTGGCGGCCGCATGCCGTCTGAA	This study
tfpC-1	AAATTAATTAAATGAAATCAAAACTCCCCTTAATCC	This study
tfpC-2	AAATTAATTAAAATCAGCAGCCATCAGGGAG	This study
tfpC-3	AAAGTTTAAACTTAGTGGTGGTGGTGATGATGCATACGTCCCAATTCTCTTTGCAG	This study
tfpC-4	AAAGTTTAAACTTACTTGTCATCGTCGTCCTTGTAGTCCATACGTCCCAATTCTCTTTGCAG	This study
tfpC-5	AAAGTTTAAACGCCTTACATACGTCCCAATT	This study
pilRBS	GGCTTTCCCCTTTCAATTAGGAG	39
SP3A	CCGGAACGGACGACCCCG	39
USS2	TGAACCAACTGCCACCTAAGG	43
pilAREV	GGGCGGCAGTGTCGAAAATTGTCAGTTTTAGTGC	This study
pilCfor	GGCGGAGGTGGCGGGGCC	45
pilCdownstream	CCATCTTTGGCGGTACCCTCGCTG	45
LS1	AATGGTGGTCAGGCAGTT	This study
LS2	TTTATCATCATCGTCAACATGATG	This study
779F	AACGACGCAGGCCATAAA	This study
779R	TTGCTGATGCCTTCGAGATAG	This study
780F	AGACGGACAGTTTGCAGAATA	This study
780R	GGCAGACCGAATCCTTATGT	This study
781F	GACCATCTGCCCAATCCTTT	This study
781R	TTTCCAGCGACAGGGTAATG	This study
782F	CGCAAGCCTCCATATACCATT	This study
782R	CCCTGATGGCTGCTGATTT	This study
783F	TAACAGCAGACGCTCCATTC	This study
783R	GCCAGACGTGCTTGTGATA	This study
784F	TGGGATAAGTTGGGCGATTT	This study
784R	TTGTACGTGTAGCCGGTATTG	This study
785F	ATACGCCCAATGCCCAAT	This study
785R	CTGCTGCTGATATTGTCTGTTTG	This study
786F	CGGGTCAAAGTCGTCATTCT	This study
786R	TTGAGTAAAGACGGCGGTATG	This study

### Constructing the parental strain N-1-60.

The pilin was unable to undergo antigenic variation due to four mutations (in the wild type, 5′-CCC CAC CCA ACC CAC CC-3′; in the multisite G4 mutant, 5′-CCC CAC CAC ACC CCC AC-3′ [from Lauren Prister]) in the guanine quadruplex site upstream of the *pilE* gene (40, 41). This mutant G4 sequence was introduced by synthesizing an ∼800-bp gBlock (Integrated DNA Technologies). The gBlock consisted of a DUS12 sequence and the multisite G4 substitutions flanked by regions of homology to the G4-*pilE* locus (479 bp on the 5′ end and 303 bp on the 3′ end). This DNA was used to spot transform FA1090. Several dilutions of the transformation reaction mixture were spread onto GCB agar plates without antibiotics and grown for 41.5 h at 37°C in the presence of 5% CO_2_. Colonies that had a piliated colony morphology (domed surface and no blebbing from the edges) were chosen and restreaked to confirm the piliated colony morphology. Cells that successfully recombined the multisite G4 mutations were identified by screening the piliated clones in pools by PCR. Briefly, clones were individually stored in glycerol at −80°C, and pools of 10 clones were tested by using a primer, multimutG4_2, that anneals only to G4 sites that carry the desired mutations, paired with RTG4-3R (42), which binds in the beginning of the *pilE* locus. Positive pools were recreated by PCR using individual clones as the templates; then the promoter was amplified and sequenced with USS2 (43) and pilAREV, and the *pilE* locus was amplified and sequenced with PilRBS and SP3A (39). This strain was the recipient in a transformation reaction with an ∼950-bp gBlock (synthesized by Integrated DNA Technologies) carrying a DUS12 sequence and the phase variation locked *pilC1*_PL_ allele (11, 44), which maintains the *pilC1* gene in a phase “on” conformation, which was flanked by 471 bp and 463 bp of homology on the 5′ and 3′ ends of DUS12*-pilC1*_PL_, respectively, at the *pilC1* locus. Dilutions of the transformant were grown on GCB plates and grown for 63.5 h at 37°C in the presence of 5% CO_2_. PCR was used to screen pools of clones that formed nonblebbing, piliated colonies before individual clones were confirmed by amplifying and sequencing *pilC1*_PL_ using the pilCfor and pilCdownstream primers (45). The resultant strain is the FA1090 multisite G4 mutant 1-81-S2 *pilE* variant *pilC1*_PL_ (N-1-60).

### NGO0783 Δ*tfpC mutant* construction.

An ∼650-bp fragment containing 270 bases upstream of the *tfpC* open reading frame, the first 30 bases of TfpC, a PacI restriction site, a hemagglutinin (HA) tag, a NotI restriction site, the last 60 bp of *tfpC*, and 99 bp downstream of *tfpC*, which included a 12-mer DNA uptake sequence (DUS12), was synthesized and cloned into the pTwist-Amp-MC vector by Twist Biosciences (*tfpC*::PacI-His-NotI) (Table 4). A PacI- and DUS12 NotI-flanked *nptII* fragment from pBSL86 (ATCC) was generated by two PCRs; the first used primers PacI_nptII181_F and DUS12_nptIIR, and the second PCR included the NotI restriction site using primers PacI_nptII181_F and NotI_DUS12. This fragment was introduced into the PacI- and NotI-digested plasmid from Twist Biosciences in between the upstream and downstream sequences of *ngo783* and in-frame. This plasmid pTwist-Δ*ngo783*::*kan* was used to spot transform several N. gonorrhoeae parent strains to generate Δ*tfpC* strains. Transformants were selected on GCB Kan and checked by diagnostic PCR and sequencing.

### Transformation efficiency assay.

The efficiency of N. gonorrhoeae transformation was performed using a protocol similar to that used in reference 46, except that 50 ng of pSY6 DNA was used instead of 150 ng. After a 20-min incubation of the cells and DNA at 37°C, 1 U DNase I was added to the transformation reaction mixtures and they were incubated for 10 min at 37°C. Transformation efficiencies are reported as the means from five independent experiments.

### Construction of tagged TfpC.

The *tfpC* ORF was PCR amplified from FA1090 genomic DNA using the following primer pairs: tfpC-1 and tfpC-3 (His tag), tfpC-1 and tfpC-4 (Flag tag), and tfpC-1 and tfpC-5 (no tag). The *tfpC* fragment included the ORF, and 244 bp upstream of the ORF was also PCR amplified using the primer pairs as follows: tfpC-2 and tfpC-3 (His tag) and tfpC-2 and tfpC-5 (no tag). The PCR products were column purified using a PCR purification kit (Qiagen), cut by PacI and PmeI, and cloned into the PacI/PmeI-digested pGCC4 (47) or pGCC2 (48, 49) vector, respectively.

The resulting isopropyl-d-1-thiogalactopyranoside (IPTG)-inducible pGCC4 construct (1 to 2 μg) was spot transformed into the parent (N-1-60) and Δ*tfpC* mutant (N-3-3). The IPTG-inducible pGCC4 construct was also used to transform FA1090 1-81-S2 *recA6* Myc-tagged *pilE* (K-16-47) and the isogenic Δ*tfpC* mutant (Q115) and FA1090 1-81-S2 Myc-tagged *pilE* (Q155) and the isogenic Δ*tfpC* mutant (Q165). Strain Q155 was constructed by using a Myc-tagged *pilE* plasmid construct to transform FA1090 (32). The pGCC2 construct was spot transformed into the parent (N-1-60) and Δ*tfpC* mutant (N-3-3). The transformants were selected on GCB with Erm and their sequences confirmed.

### *pilT* mutant construction.

*pilT* mutants were constructed by using 1 μg of FA1090 Δ*pilT*::*erm* genomic DNA (50) in spot transformations into the parent, namely, FA1090 1-81-S2 *myc*-tagged *pilE* (Q155), and the isogenic Δ*tfpC* mutant (Q165) and selected on GCB Erm plates.

### Western blot analysis.

Colonies grown on GCB with 0.0, 0.025 mM, or 0.1 mM IPTG for 22 h were swabbed into phosphate-buffered saline (PBS) buffer, and the resuspensions were directly protein quantitated using a Pierce bicinchoninic acid (BCA) protein assay kit (ThermoFisher). Twenty-five micrograms of total protein per lane was run on a 4 to 15% SDS-PAGE gels (Bio-Rad) at 150 V and transferred to Immobilon-P membranes at 250 mA. A replicate gel was run and stained with Coomassie brilliant blue to analyze total protein loading per lane. The blot was blocked in 5% nonfat milk in TBST (TBS plus 0.1% Tween 20) overnight. Anti-c-Myc antibody (Sigma) or anti-Flag antibody (Rockland) was diluted 3,000× in TBST to detect the Myc-tagged PilE or Flag-tagged TfpC on a shaker for 1 h at room temperature, respectively. The blot was washed 6 times with TBST for 5 min each time and then incubated with a 20,000×-diluted secondary antibody, peroxidase-conjugated goat anti-rabbit IgG (H+L) (Jackson ImmunoResearch) for 1 h. After secondary antibody binding and subsequent washing, the blot was analyzed using an ECL Prime detection kit (GE Healthcare). After enhanced-chemiluminescence (ECL) detection of Myc-PilE, the same blot was washed 5 times for 10 min in TBST using a large volume of wash buffer, blocked for 1 h, and subjected to immuno-detection using anti-RecA (E. coli) antibody (3,000× dilution) (gift from Mike Cox [51]) and analyzed using an ECL Prime detection kit (GE Healthcare). Densitometry was performed using ImageJ (https://imagej.nih.gov/ij/).

### Immuno-transmission electron microscopy.

For analysis of piliation on strains grown on solid medium, immunoelectron microscopy was performed as described previously (52). Briefly, Formvar/carbon-coated copper grids were used to lift cells directly from 18-h-old colonies and fixed for 15 min by addition of a drop (17 μl) of 0.2% glutaraldehyde and 4% paraformaldehyde in Dulbecco’s PBS (DPBS; Fisher) onto the grids. The grids were washed 3 times with 1% bovine serum albumin (BSA; Sigma) in DPBS and blocked in 0.1% gelatin (Aurion, Inc.) in DPBS for 30 min. The grids were washed once with 1% BSA in DPBS and incubated with a 1:10 dilution of rabbit anti-c-Myc antibody (Sigma) for 1 h. The grids were washed three times with 1% BSA in DPBS and incubated with 0.1% gelatin in PBS for 30 min. They were then washed once with BSA in DPBS and incubated with goat anti-rabbit IgG antibody conjugated to 12-nm gold particles (1:20 dilution; Jackson ImmunoResearch Laboratories) for 1 h. The grids were washed five times in water for 3 min each and then negatively stained with 1% uranyl acetate for 1 min. All washes and incubations were with 17 μl and performed at room temperature. The liquid on the grids after each step was carefully wicked away using a Whatman paper. Grids were viewed using an FEI Tecnai Spirit G2 transmission electron microscope.

### Imaging of pilus-dependent colony morphology.

Representative colonies after 22 h of growth on solid medium were observed and recorded using a Nikon SMZ-10A stereomicroscope and a Nikon digital-sight camera.

### Quantitative RT-PCR.

Overnight colonies on plain GCB plates were resuspended in GCB with 5 mM sodium bicarbonate and adjusted to an optical density at 600 nm (OD_600_) of ∼0.15, grown at 37°C on a rotor for 3 h, and treated with different concentrations of IPTG for 1 h. The cells were treated with 2 vol of RNA Protect bacterial reagent (Qiagen) and then collected by centrifugation at 4,000 rpm for 5 min. Total RNA was isolated using a RNeasy minikit (Qiagen) and treated with RQ1 DNase (Promega) to remove genomic contamination. The quantitative RT-PCR was performed as described before (53). The 783f and 783r primer pair was used to determine the expression of TfpC with increasing IPTG concentrations. The following primer pairs were used to detect the effect of the Kan insertion into the Δ*tfpC* mutant on the surrounding genes in the operon: (i) 779f and 779r, (ii) 780f and 780r, (iii) 781f and 781r, (iv) 782f and 782r, (v) 784f and 784r, (vi) 785f and 785r, and (vii) 786f and 786r (Table 4).

### Cloning, expression, and purification for NMR.

DNA encoding full-length TfpC (residues 1 to 147), minus the region encoding the N-terminal periplasmic signal sequences, was synthesized by Synbio Technologies and cloned into the pET28b vector using NcoI and XhoI restriction sites (Table 4). A variant encoding N-terminally truncated TfpC (TfpC-CTD; residues 52 to 147) was created by deletion PCR with primers LS1/LS2 (Table 4) using the pET28b*tfpC* plasmid as a template. Expression was carried out in E. coli BL21(DE3) cells (New England Biolabs), where cells were grown in the presence of 50 μg/ml kanamycin at 37°C in M9 minimal medium supplemented with ^15^NH_4_Cl (Sigma). Expression was induced with 0.5 mM IPTG at an OD_600_ of 0.6, and cells were harvested after growth overnight at 18°C. Cells were resuspended in 20 mM Tris-HCl (pH 8)–200 mM NaCl, lysed by sonication, and purified using nickel affinity chromatography (Qiagen). Samples were then gel filtered using a Superdex 200 column (GE Healthcare) equilibrated in 20 mM Tris-HCl (pH 8)–200 mM NaCl.

### NMR spectroscopy.

NMR measurements were performed on 0.25 mM ^15^N-labeled samples of TfpC and TfpC-CTD in 50 mM NaPO_4_ (pH 6.0)–100 mM NaCl–1 mM tris(2-carboxyethyl)phosphine–10% D_2_O and 50 mM NaPO_4_ (pH 6.0)–100 mM NaCl–10% D_2_O, respectively. Two-dimensional (2D) ^1^H-^15^N HSQC experiments were recorded with 32 scans at 298 K on a Bruker Avance III HD 700 spectrometer, equipped with a TCI cryoprobe. Data were processed using NMRPipe (54) and analyzed using NMRViewJ (55).

### Structural modeling.

Signal peptide analysis was carried out using SIGNALP (56), and secondary-structure and domain analyses were performed using PSIPRED (57). Coevolution analysis of mature TfpC (residues 1 to 147) was carried out using the EVcouplings Python framework (34), using default parameters. One thousand sixty-five homologous sequences were identified and used in the initial alignment stage (at an effective sequences-to-protein length ratio of 5.15) and yielded 77 strong evolutionary couplings. These couplings were then used as interresidue distance restraints to guide modeling of the TfpC structure within the EVcouplings Python framework. Models where the N-terminal region was folded back into the C-terminal region were discarded. The final model had a ranking score of 0.75 and was representative of the highest cluster of models.
